# Measuring Global Trends in the Status of Biodiversity: Red List Indices for Birds

**DOI:** 10.1371/journal.pbio.0020383

**Published:** 2004-10-26

**Authors:** Stuart H. M Butchart, Alison J Stattersfield, Leon A Bennun, Sue M Shutes, H. Resit Akçakaya, Jonathan E. M Baillie, Simon N Stuart, Craig Hilton-Taylor, Georgina M Mace

**Affiliations:** **1**BirdLife InternationalCambridgeUnited Kingdom; **2**Applied Biomathematics, SetauketNew YorkUnited States of America; **3**Institute of Zoology, Zoological Society of LondonLondonUnited Kingdom; **4**CI/CABS-IUCN/SSC Biodiversity Assessment Unit, Center for Applied Biodiversity Science, Conservation InternationalWashington, District of ColumbiaUnited States of America; **5**IUCN Red List Programme, IUCN/SSC United Kingdom OfficeCambridgeUnited Kingdom

## Abstract

The rapid destruction of the planet's biodiversity has prompted the nations of the world to set a target of achieving a significant reduction in the rate of loss of biodiversity by 2010. However, we do not yet have an adequate way of monitoring progress towards achieving this target. Here we present a method for producing indices based on the IUCN Red List to chart the overall threat status (projected relative extinction risk) of all the world's bird species from 1988 to 2004. Red List Indices (RLIs) are based on the number of species in each Red List category, and on the number changing categories between assessments as a result of genuine improvement or deterioration in status. The RLI for all bird species shows that their overall threat status has continued to deteriorate since 1988. Disaggregated indices show that deteriorations have occurred worldwide and in all major ecosystems, but with particularly steep declines in the indices for Indo-Malayan birds (driven by intensifying deforestation of the Sundaic lowlands) and for albatrosses and petrels (driven by incidental mortality in commercial longline fisheries). RLIs complement indicators based on species population trends and habitat extent for quantifying global trends in the status of biodiversity. Their main weaknesses are that the resolution of status changes is fairly coarse and that delays may occur before some status changes are detected. Their greatest strength is that they are based on information from nearly all species in a taxonomic group worldwide, rather than a potentially biased subset. At present, suitable data are only available for birds, but indices for other taxonomic groups are in development, as is a sampled index based on a stratified sample from all major taxonomic groups.

## Abstract 2

An index is developed from the IUCN Red List that measures the overall threat status of taxa and reveals that the status of the world's birds has deteriorated since 1988

## Introduction

The world's biodiversity is diminishing rapidly (Balmford et al. 2003; Jenkins et al. 2003). At the 2002 World Summit on Sustainable Development, the nations of the world agreed to pursue more effective implementation of the objectives of the Convention on Biological Diversity (CBD) in order to achieve a significant reduction in the current rate of loss of biological diversity by 2010 (Secretariat of the Convention on Biological Diversity 2003).

The European Union has adopted the more ambitious target of halting the loss of biodiversity by 2010 (European Union 2001). We do not yet have an adequate way of monitoring progress towards achieving this target. However, the CBD Conference of the Parties at its Seventh Meeting adopted Decision VII/8, which included recommendations to develop indicators for measuring trends in the components of biodiversity based on (1) trends in extent of selected biomes, ecosystems, and habitats; (2) trends in abundance and distribution of selected species; (3) change in status of threatened species; (4) trends in genetic diversity of domesticated animals and cultivated plants; and (5) coverage of protected areas (CBD 2004). None of these alone is adequate, but together they provide powerful measures of global trends in the status of biodiversity.

Here we address the third of this suite of indicators, using changes in the threat status of species as measured by the categories of the World Conservation Union (IUCN) Red List. The IUCN Red List is widely recognised as the most objective and authoritative listing of species that are globally at risk of extinction (Lamoreux et al. 2003; Hambler 2004). Species are assigned to Red List categories (see Abbreviations section) through detailed assessment of information against a set of objective, standard, quantitative criteria (IUCN 2001; Table 1). Thousands of scientists, many of whom are members of IUCN Specialist Groups and the IUCN Species Survival Commission network, provide extensive information for assessments. Over the last few years, the IUCN Red List has been developed into a global programme to monitor the extent and rate of biodiversity degradation. The programme is currently overseen by four partner organisations: the IUCN Species Survival Commission, BirdLife International, NatureServe, and the Center for Applied Biodiversity Science at Conservation International, with additional partners being recruited, in particular to provide plant and marine expertise. Red List Authorities are appointed to ensure consistent categorisation between species and groups and for organising independent scientific review. A Red List ‘Standards and petitions' subcommittee monitors the process and resolves challenges and disputes to listings.

**Table 1:**
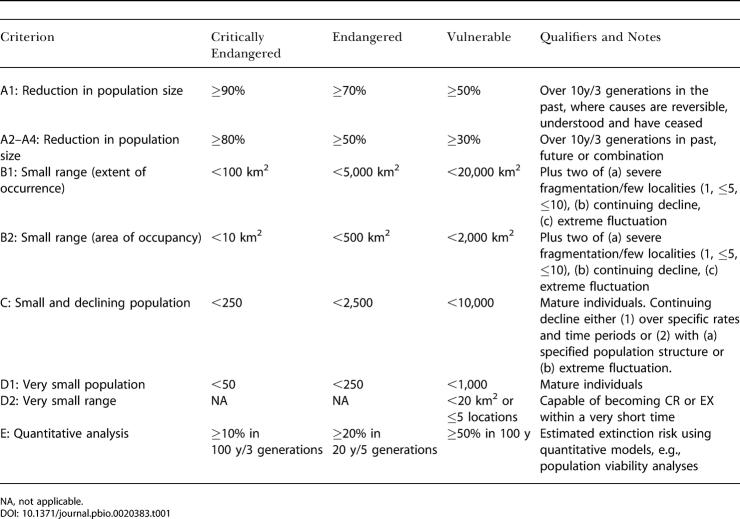
Simplified Overview of Thresholds for the IUCN Red List Criteria

NA, not applicable

One of the goals of the programme is to provide a global index of the changing state of biodiversity (IUCN 2004). However, previous attempts to use the IUCN Red List to provide answers about the rate of loss of biodiversity suffered from many limitations (Cuarón 1993; Smith et al. 1993), and a number of problems with the general approach have been identified (Burgman 2002; Possingham et al. 2002; but see Lamoreux et al. 2003). We show, using data from birds, how the limitations can be overcome, and we present for the first time Red List Indices (RLIs) that are robust, temporally sensitive, representative, and comprehensive. These provide unique baseline data on the rate of loss of biodiversity against which progress towards meeting the CBD 2010 target can be judged. They also allow finer-scale resolution of trends in particular biogeographic realms, ecosystems, and habitats.

## Results

**Figure 1:**
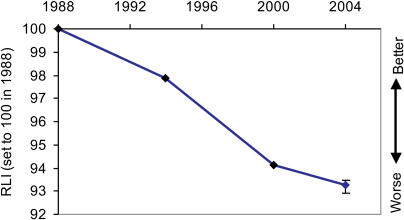
The RLI for All Bird Species Sample size: 250 genuine status changes/2,469 species in categories Extinct in the Wild to Near Threatened in at least one assessment. Error bars for 2004 RLI value based on estimated number of genuine status changes for 2000–2004 not yet detected owing to information time lags (see Materials and Methods for further details).

**Figure 2:**
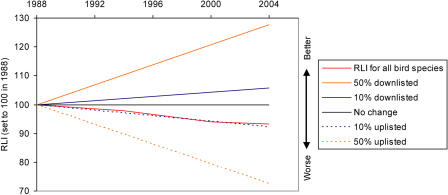
The RLI for All Bird Species in 1988–2004 Compared to Hypothetical Indices Hypothetical indices show trends if no species had changed category, and if 10% or 50% of species in the categories from Near Threatened to Critically Endangered had been uplisted to a higher category of threat or downlisted to a lower category of threat over the period.

The total number of extant threatened and Near Threatened birds listed on the IUCN Red List has changed relatively little over the four complete assessments of all the world's birds, increasing from 1,664 species in 1988 to 1,990 species in 2004. However, large numbers of species have moved between categories, particularly in the earlier assessments (Table 2). Most of these category changes have been a consequence of improved knowledge (including improved consistency of interpretation of information against the Red List criteria) or revised taxonomy. However, a significant proportion of species (equating to 2.4%–7.3% of threatened or Near Threatened species in each assessment) have moved between categories because of genuine improvement or deterioration in status. The RLI for birds illustrates the combined effect of these genuine status changes, to provide a simple metric of the changing overall status of the world's birds, in terms of their relative projected extinction risk as estimated using the categories of the IUCN Red List. This shows that there has been a steady and continuing deterioration in the threat status of the world's birds between 1988 and 2004, with an overall change in the index value of −6.90% over this period (Figure 1; see Table 2). No change would indicate that the average status of all bird species was the same as in 1988. To put this into context, if 10% of species in the categories from Near Threatened to Critically Endangered had deteriorated in status sufficiently to be uplisted one category to a higher category of threat between 1988 and 2004, the index would have changed by −7.8%, and if 50% of such species had deteriorated by one category the index would have changed by −27.4% (Figure 2). The error bars for the 2004 RLI value (based on the projected number of genuine status changes for the 2000−2004 period yet to be detected owing to information time lags; see Discussion) show that the estimated recent RLI trends are likely to be fairly robust.

**Table 2:**
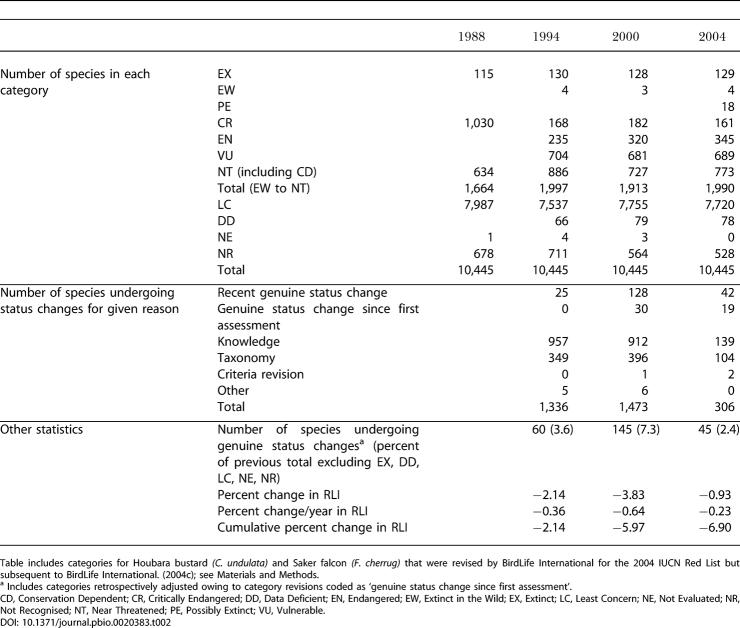
Number of Species in Each IUCN Red List Category as Published in Collar and Andrew (1988), Collar et al. (1994), BirdLife International. (2000, 2004c), and the Number of Species Undergoing Genuine Status Changes in Each Period

Table includes categories for Houbara bustard *(C. undulata)* and Saker falcon *(F. cherrug)* that were revised by BirdLife International for the 2004 IUCN Red List but subsequent to BirdLife International. (2004c); see Materials and Methods

^a^ Includes categories retrospectively adjusted owing to category revisions coded as ‘genuine status change since first assessment'

CD, Conservation Dependent; CR, Critically Endangered; DD, Data Deficient; EN, Endangered; EW, Extinct in the Wild; EX, Extinct; LC, Least Concern; NE, Not Evaluated; NR, Not Recognised; NT, Near Threatened; PE, Possibly Extinct; VU, Vulnerable

**Figure 3:**
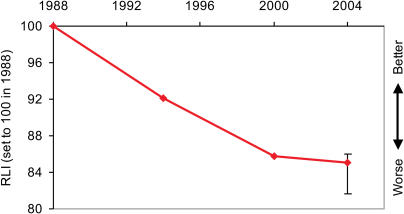
The RLI for All Bird Species with Categories Weighted by Relative Extinction Risk Sample size: 250 genuine status changes/2,469 species in categories Extinct in the Wild to Near Threatened in at least one assessment. Error bars for 2004 RLI value based on estimated number of genuine status changes for 2000–2004 not yet detected owing to information time lags (see Materials and Methods for further details).

To examine trends in the status of the most threatened species (i.e., those closest to extinction), the index was calculated using weights for each category related to the relative extinction risk associated with them (Figure 3; see Materials and Methods). This shows a levelling out of the decline in the index value during 2000–2004 (although the error bars indicate that in the next few years the belated discovery of genuine status changes for this period could reduce this apparent levelling out). This was because for those species that underwent genuine status changes in the categories of highest extinction risk (those that have the greatest influence on the index value when calculated in this way), the number of species that deteriorated in status during this period was balanced by the number that improved in status owing to conservation action. Specifically, two Critically Endangered species became Extinct (or Possibly Extinct) in the wild (Hawaiian crow [Corvus hawaiiensis] and Spix's macaw [Cyanopsitta spixii]), and five Endangered species became Critically Endangered, but this was offset by seven species that improved in status as a result of conservation efforts (including, e.g., Polynesian megapode [Megapodius pritchardii], Christmas Island hawk-owl [Ninox natalis], and Christmas Island white-eye [Zosterops natalis]; BirdLife International 2004c).

**Figure 4:**
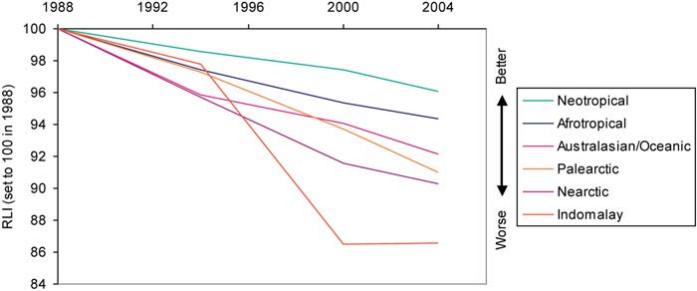
RLIs for Birds in Different Biogeographic Realms Sample sizes: Neotropical, 49 genuine status changes/834 species in categories Extinct in the Wild to Near Threatened in at least one assessment; Afrotropical, 41/394; Australasian/Oceanic, 53/614; Palearctic, 34/238; Nearctic, 9/92; Indo-Malayan, 100/585.

**Figure 5:**
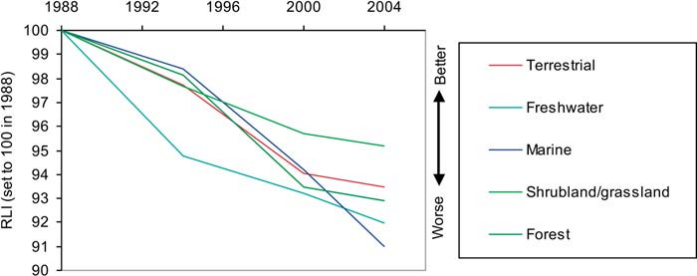
RLIs for Birds in Different Habitats Sample sizes: terrestrial, 206 genuine status changes/2,329 species in categories Extinct in the Wild to Near Threatened in at least one assessment; freshwater, 31/226; marine, 12/133; shrubland/grassland, 45/481; forest, 169/1,513.

**Figure 6:**
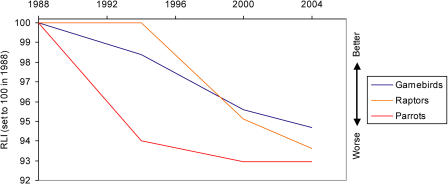
RLIs for Three Bird Families with High Conservation Profiles Sample sizes: game birds, 15 genuine status changes/123 species in categories Extinct in the Wild to Near Threatened in at least one assessment; raptors, 10/93; parrots, 19/148.

**Figure 7:**
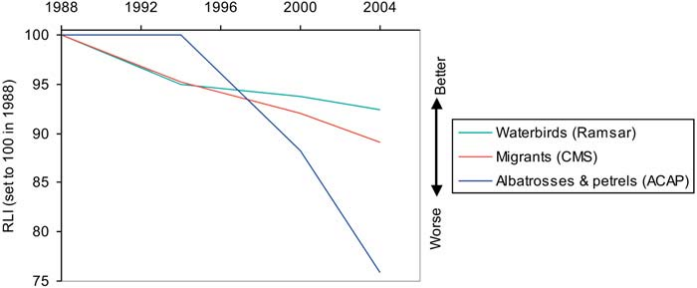
RLIs for Three Species Groups Targeted by Particular International Conservation Treaties: The Ramsar Convention on Wetlands, the CMS, and the ACAP under the CMS Sample sizes: waterbirds, 36 genuine status changes/238 species in categories Extinct in the Wild to Near Threatened in at least one assessment; albatrosses and petrels, 6/28; migrants, 50/313.

The RLI can be broken down by biogeographic realm (Figure 4), ecosystem, habitat type (Figure 5), and for particular species groups (Figures 6 and 7). These show that the threat status of birds has deteriorated worldwide, with a more-or-less similar rate and proportional extent of deterioration in the Nearctic, Neotropical, Palearctic, Afrotropical, and Australasian/Oceanic realms. The RLI for the Indo-Malayan realm showed a steeper rate of decline during the 1990s (see Figure 4). Declines in the index for three major ecosystems (terrestrial, freshwater, and marine) and two terrestrial habitat types (forest and shrubland/grassland) all show a broadly similar pattern (see Figure 5), although the declines in the freshwater environment appear to have been the most severe. Finally, RLIs for selected subsets of species (including those relevant to particular international treaties) highlight the severity of the worsening situation of the world's albatrosses and large petrels in recent years (see Figures 6 and 7).

## Discussion

### How Fast Are We Losing Avian Biodiversity?

Bird species are being driven Extinct by increasing human impacts on the planet. In total, 129 bird species have been classified as Extinct since 1500, with an additional four species listed as Extinct in the Wild, but surviving in captive populations (BirdLife International 2004b, 2004c). Additionally, 18 Critically Endangered species are considered Possibly Extinct by BirdLife International. (2004b, 2004c; see Materials and Methods). Of these confirmed and likely extinctions, nine have occurred during the period 1988–2004 (BirdLife International, unpublished data). However, it is very difficult to produce robust estimates of recent extinction rates and to quantify how they have changed over short timescales. This is because extinction is difficult to detect once species become very rare (Diamond 1987; Reed 1996), and tiny populations of species potentially doomed by habitat loss or other threats may persist for many decades (Turner 1994; Brooks et al. 1997, 1999). For these reasons, extinction rates perform weakly as indicators of the current state of biodiversity (Balmford et al. 2003).

By contrast, the RLIs presented here provide a robust, sensitive measure of the rate at which the world's birds are changing in relative projected extinction risk, as classified using the categories of the IUCN Red List. The indices show that the overall threat status of the world's birds has deteriorated steadily over the last 16 y. The RLI value has changed by −6.90% over this period. However, it should be noted that owing to the arbitrary nature of the weights applied to each category to calculate the score, this percentage decline is not directly comparable with percentage declines reported for population-based indices such as the Living Planet Index (Loh 2002) or the United Kingdom headline indicator for wild bird populations (Gregory et al.2003).

When the RLI is weighted by the relative extinction risk associated with each category in order to emphasise trends in the status of the most threatened species, the rate of decline of the index value appears to have levelled off in recent years (see Figure 3), owing to the number of such species deteriorating in status being balanced by the number improving. Nevertheless, it should be emphasised that one Critically Endangered species went Extinct in the Wild in the wild during the period (Hawaiian crow [C. hawaiiensis]), and another is highly likely to have done so (Spix's macaw [C. spixii]; BirdLife International 2004c). These are potentially irretrievable losses to genetic diversity.

How can we interpret the RLI in relation to the CBD's target of reducing the rate of loss of biodiversity by 2010? The interpretation is different for measures of the state of biodiversity (e.g., total area of remaining forest) and measures of the rate of change in this state (e.g., annual percentage forest loss). For indices based on proportional change in a measure (plotted on a negative scale as with the RLI), if the measure is one of state, a significant diminution in downward trend would show that the target has been met. If the measure is one of rate of change of state, however, the target is not met until we see a positive trend, not just a decelerating decline. Some of the Red List criteria are based on absolute population size or range size, while others are based on rates of decline in these values or combinations of absolute size and rates of decline. These criteria are used to assign species to Red List categories that can be ranked according to relative projected extinction risk, and the RLI is calculated from changes between these categories. Hence an RLI value relates to the rate at which species are slipping towards extinction at a particular time. To show that the 2010 target has been met, the RLI must therefore show a positive trend. A downward trend, even if diminishing, shows that the slide of species towards extinction is accelerating, not slowing down. The negative trends in the RLI values (see Figure 1) thus show that in 2004 we are losing biodiversity at an increasing rate.

The RLIs show some interesting regional variations. The index for birds in the Indo-Malayan realm shows a sharp decline during the 1990s (see Figure 4). This was a result of the intensifying destruction of forests in the Sundaic lowlands of Indonesia, which escalated particularly in the late 1990s and led to predictions of almost total loss of lowland forest in Sumatra by 2005 and in Kalimantan by 2010 (Holmes 2000; BirdLife International 2001). As a consequence of these increasing rates of habitat loss, many species were uplisted to higher categories of threat under criterion A (rapid population declines). However, it is notable that there has been a significant deterioration in the threat status of birds of shrubland/grassland habitats as well as forest, and in the two other major ecosystems (freshwater and marine), indicating that birds in a broad spectrum of environments are under threat.

RLIs can be calculated for particular species groups that have specific conservation or policy significance. For example, there are particularly active conservation networks for game birds (e.g., World Pheasant Association), raptors (e.g., World Working Group on Birds of Prey), and parrots (e.g., Loro Parque and World Parrot Trust), and the threat status of all three of these species groups is deteriorating, with steeper declines in the index value for parrots in the earlier part of the period (see Figure 6). In addition, there are several international conservation treaties targeting particular suites of species (the Ramsar Convention on Wetlands, the Convention on Migratory Species [CMS], and the Agreement on the Conservation of Albatrosses and Petrels [ACAP] under the CMS) for which disaggregated RLIs provide a metric against which to judge their success in improving the fortunes of the species involved. The RLI for albatrosses and large petrels shows how dramatically their threat status has deteriorated in recent years (see Figure 7). This is closely linked to the expansion of commercial longline fisheries (both legal and illegal), which causes incidental mortality of albatrosses and other seabirds when they get caught on baited hooks and drown (Tuck et al. 2001; 2003; BirdLife International 2004b). The total reported effort from fleets in the southern oceans has been well over 250 million hooks per year since the early 1990s, with some fleets expanding rapidly in the last decade (Tuck et al. 2003). Models for at least some albatross species show clear links between population declines and these increases in longline fishing effort (Tuck et al. 2001). Mitigation measures are effective (Løkkeborg 2001), and the RLI will provide a useful measure by which to judge the effectiveness of the implementation of ACAP, following its entry into force in 2004.

It should be noted that setting all disaggregated index values to a common baseline in 1988 obscures any changes prior to this period (see, e.g., Pauly 1995). For example, although the Indo-Malayan realm has shown the most severe recent index declines, ‘only' six extinctions occurred there between 1500 and 1988, whereas at least 62 bird species are known to have gone Extinct in the Australasian/Oceanic realm during the same period, and 40 in the Afrotropical realm (BirdLife International 2000). Similarly, the terrestrial ecosystem has suffered far more extinctions since 1500 (115) than the freshwater (17) or marine ecosystems (five), but all are set to a common baseline in 1988.

### Category Weights

The RLI is based on the number of species in each Red List category. In order to make the index sensitive, not just to the total number of threatened species, but also to the changes in category assigned to each species, each category was given a weighting. We used an ‘equal-steps' approach (with incremental increases from one for Near Threatened through to five for Extinct) to reflect the ordinal ranks of the categories, whereby each step from Least Concern to Extinct indicates that at least one measure of extinction risk has become worse. The advantage of this approach is that it is simple, and the trends in the resulting index are driven by a relatively large number of species (hence producing a more robust and representative index). This is because a species moving from Least Concern to Near Threatened contributes just as much to the changing score as a Critically Endangered species going Extinct, and the numbers of species in each category (and the number moving in and out of each category) increases disproportionately from Critically Endangered to Least Concern (see Table 2).

However, steps between lower categories of threat represent smaller increases in extinction risk than steps between higher categories. Therefore we also tested an ‘extinction risk' approach, with each category weighted according to its relative extinction risk based on the quantitative thresholds for each of its criteria (Table 3). Although this approach also relies on some assumptions (e.g., about the type of extinction risk curve, and the extinction risk associated with Near Threatened), it is based on the principles of extinction dynamics, in contrast to the equal-steps approach.

**Table 3:**
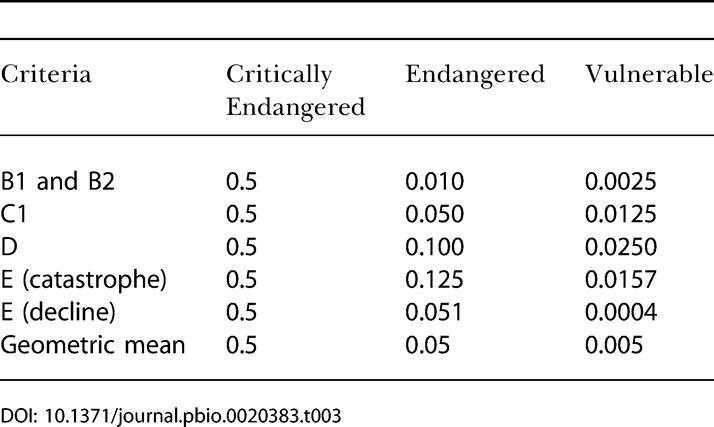
Weights for Red List Categories Critically Endangered, Endangered, and Vulnerable, Based on Relative Extinction Risk Associated with Various Red List Criteria

The most important difference between the two approaches is the effect of status changes in less-threatened or nonthreatened species. The equal-steps approach gives an index that is heavily influenced by movements of species among the lower categories of threat. The extinction risk approach gives an index that is largely influenced by movements of species among the higher threat categories. For example, if a Vulnerable species improves in status and becomes Near Threatened, and at the same time, a Critically Endangered species goes Extinct, the RLI based on equal-steps weights registers no change, but the index based on extinction risk weights shows a substantial decrease. Downlisting of a Vulnerable species to Near Threatened might represent a very substantial population increase, whereas extinction of a Critically Endangered species might represent the loss of very few individuals. The latter is arguably more significant in terms of genetic diversity, but the former might be more important as an indicator of wider biodiversity trends. Thus, the extinction risk weights emphasise the loss of biodiversity owing to imminent or potential extinctions of species, whereas the equal-steps weights allow the index to capture large changes in the populations of less-threatened species.

For the RLI for complete taxonomic groups, and for disaggregating the index to show trends for subsets of species, for example, in particular realms or ecosystems we used the equal-steps approach because the number of species moving between the higher threat categories (those effectively driving trends when an ‘extinction risk' weighting is used) was too small to be meaningful in disaggregated indices. Only 23% of all genuine status changes (58 species in total) involved moves in or out of the highest threat categories. However, for examining trends in the species closest to extinction, we used the weights based on relative extinction risk.

### Weaknesses of RLIs

The usefulness of the IUCN Red List as an indicator of trends in the status of biodiversity (e.g., Smith et al. 1993) has been previously questioned on the grounds that (1) the categories are subjective; (2) taxonomic treatment is uneven, and listings are biased towards attractive, spectacular, high-profile, or better-known species; and (3) most species move between categories because of changes in knowledge or taxonomy, not as a consequence of genuine improvement or deterioration in status (Cuarón 1993; Burgman 2002; Possingham et al. 2002; but see Lamoreux et al. 2003).

The first of these problems has been addressed since 1994, when quantitative and objective categories and criteria for the IUCN Red List were introduced (IUCN 1994, 2001). The second problem can be overcome by calculating indices only for taxonomic groups in which all species have been comprehensively assessed and reassessed (as shown here) or by developing indices based on a stratified sample from diverse taxonomic groups (see below). The third problem has already been addressed because since 2001 the IUCN Red List has required clear documentation of the reason for any reclassification (IUCN 2001). Hence, movements of species between categories owing to knowledge, taxonomy, or other ‘nongenuine' reasons can be easily excluded when calculating the index.

RLIs have a fairly coarse level of resolution of status changes because of the broad nature of Red List categories. Populations in the wild may have to undergo quite significant changes in size, trend, or distribution before crossing the thresholds to qualify for a higher or lower Red List category and, hence, before changing the RLI value. This is inherent in using the Red List categories rather than more precise parameters such as estimates of population size. It is not always true, however: The Red List criteria allow for species to be assessed as threatened on the basis of projected declines, and thus changes in status can reflect new or emerging threats in anticipation of population or range changes. We suggest that the disadvantage of coarse resolution is outweighed by the advantage of using a system that allows all the world's species in a taxonomic group to be assessed, rather than just a (potentially biased) subset for which adequately detailed information is available.

Insensitivity of the index to status changes may also arise from time lags between changes in a species' population or range and changes in the RLI value, because of delays before detection of the status change, and/or before this knowledge becomes available to assessors. This is potentially more problematic, but several factors act to mitigate it. The Red List Programme partners have a large and expanding network of scientists across the world providing detailed and up-to-date information for an increasing number of species. Furthermore, with improving channels of communication (in particular, the increasing use of the World Wide Web to solicit information, for example, BirdLife's Web-based Globally Threatened Bird discussion forums; BirdLife International 2004a), we expect that such delays will diminish. For birds, the data support this: whereas just 42% of genuine status changes between 1988 and 1994 were detected in 1994 (with 43% detected during 1994–2000 and 15% detected during 2000–2004), 88% of changes during 1994–2000 were detected in 2000, and just 12% were detected in the subsequent 4 y. Using the data from the 1994–2000 period (because information gathering has improved considerably since 1988–1994), we can estimate the likely number of genuine status changes for 2000–2004 that have not yet been detected (six; see Materials and Methods) and, hence, estimate the possible degree of error associated with the 2004 RLI value. The results show that it may be an under- or overestimate by 0.21%–0.37% (see Figure 1): a small and acceptable margin of error. In future, we anticipate smaller retrospective adjustments to the index values, and a smaller and more predictable error associated with the most recent index value. The major advantage of backtracking status changes to the appropriate time period is that the index trends do not get distorted by the belated discovery of genuine status changes, which, for example, might result from the exhaustive research that takes place when a Red Data Book is published (e.g., BirdLife International 2001). This arguably outweighs the disadvantage that the slope of the index between two particular dates may change slightly in future releases of the index.

How robust are RLIs? A potential criticism is that they are based on status changes in small numbers of species. However, between 1988 and 2004 the RLI declined by a degree equivalent to almost 10% of species in the categories Near Threatened to Critically Endangered deteriorating in status sufficiently to be uplisted by one category to a higher category of threat. Although relatively few in number (250), these status changes are the most important among the world's birds in terms of changes in projected extinction risk. We therefore suggest that the declines shown by the RLI since 1988 represent very significant losses to global biodiversity.

Relatively large numbers of species changed categories in 1994 and 2000 owing to improvements in knowledge and improved consistency of interpretation of information against the Red List criteria (see Table 2). This was because of the introduction of quantitative criteria for assigning species to categories in 1994 (Collar et al. 1994; IUCN 1994) and the mapping of all threatened species and more rigorous justification for Near Threatened status in 2000 (BirdLife International 2000). By 2000–2004, only 6.7% of threatened and Near Threatened species changed category owing to improved knowledge (see Table 2). Nevertheless, it is true that a small proportion of species may be sufficiently poorly known that there is uncertainty over their status and whether this has changed over time. If this introduces any bias, it may be towards an overoptimistic RLI trend. This is because well-studied species (with better data and hence more certain Red List assessments) may be more likely to be those receiving conservation attention and, hence, improving in status (or at least deteriorating less rapidly). All data used in Red List assessments for birds (e.g., population size, trends, etc.) are coded for data quality, and in future the RLI will also be calculated separately for species with high-quality data, in order to test whether such biases exist.

### Strengths of RLIs

The greatest strength of the RLIs presented here is that they are based on comprehensive and complete assessments of nearly all species in a taxonomic group across the world (just 0.8% of birds are listed as Data Deficient and hence excluded from the calculation of the RLI). Most other global indicators based on, for example, population estimates, are derived from data biased towards common, well-studied species in the developed world, particularly Europe and North America. For example, the Living Planet Index (Loh 2002) is based on indices for populations in marine, freshwater, and forest ecosystems. However, 70% of the 195 populations contributing to the freshwater ecosystem are in Europe or North America, while just 18% of the 282 populations contributing to the forest ecosystem index are in the tropics, where the greatest biodiversity is found (Loh 2002). Similarly, in a global index based on data from 936 amphibian populations from 37 countries around the world, 89% of populations (835) were from Europe or North America, and just 2.2% (21) were from Asia and 5.5% (51) from South/Central America (Houlahan et al. 2000). By contrast, the RLI for birds is based on trends for nearly all the world's 10,000 bird species. RLIs for other completely assessed taxonomic groups are in development (see below).

At present, indicators based on more representative suites of species are only available for particular countries or regions, such as the United Kingdom headline indicator for birds (Gregory et al. 2003) and the Pan-European Common Bird Index (BirdLife International 2004b; Gregory et al. 2004). Indices based on population trends (particularly at the regional scale) generally include few species that are rare, localised, or difficult to survey, including those most susceptible to extinction. RLIs can incorporate status changes in such species because the Red List process is an effective system for making meaningful inferences from data that are imprecise or incomplete.

Species-based indicators such as the RLI arguably provide far more powerful measures of biodiversity loss than other indicators proposed for measuring progress towards the 2010 target (CBD 2004). Trends in the extent of biomes and habitats are of necessarily coarse resolution and take no account of the distribution of biodiversity within and between habitats; trends in the genetic diversity of domesticated animals and cultivated plants provide measures related to only a tiny proportion of biodiversity; and trends in the coverage of protected areas are a measure of responses to biodiversity loss rather than a measure of the state of biodiversity.

### Future Steps

At present, data are only available for birds to produce the sorts of indices shown here. By 2010 at least two complete global assessments will also be available, and RLIs calculated for all the world's mammals (about 5,000 species), amphibians (about 5,700 species), and hopefully some plant and marine groups. Additional indices, and an aggregation of RLI trends in multiple groups, will provide a more representative picture of the changing state of biodiversity. In recognition that this will take some time to implement, the IUCN Red List Programme is also developing a sampled RLI based on a stratified sample of about 3,000 species from all major taxonomic groups, biogeographic realms, ecosystems, and Red List categories. This will provide an index that may be more representative of trends in the threat status of all biodiversity. We suggest that RLIs will have a key role to play alongside other types of indicators in assessing progress towards reducing the rate of, or halting, the loss of biodiversity.

## Materials and Methods

### 

#### IUCN Red List assessments for birds

BirdLife International (formerly the International Council for Bird Preservation) has been responsible for providing the assessments of the world's 10,000 or so species of birds for the IUCN Red List since 1963. Since 1988, BirdLife has assessed every species of bird on a regular basis, and birds are regarded as the most comprehensively documented class of organisms on the Red List. BirdLife is the official Red Listing Authority for birds, and assessments are based on data gathered from the BirdLife Partnership of organisations in over 100 countries around the world, from published and unpublished literature, and from information provided by a worldwide network of over 1,000 species experts (BirdLife International 2000, 2004c).

The principal categories on the IUCN Red List are: Extinct, Extinct in the Wild, Critically Endangered, Endangered, Vulnerable, Near Threatened, and Least Concern (IUCN 2001). Since all bird species have been assessed, none is listed as Not Evaluated, and only 78 (0.8%) are listed as Data Deficient. In addition, two terms used by BirdLife have not yet been adopted for more general application in the IUCN Red List. Possibly Extinct is a tag applied to those Critically Endangered species that are, on the balance of evidence, ‘likely to be Extinct, but for which there is a small chance that they may still be extant, and hence they should not be listed as Extinct until local or unconfirmed reports have been discounted, and adequate surveys have failed to find the species' (BirdLife International 2004c). As there are taxonomic revisions between assessments, ‘Not Recognised' is applied to taxa in those assessments when they were not treated as full species (BirdLife International 2004c).

Data from which to calculate the indices were derived from four complete assessments of the status of the world's birds by Collar and Andrew (1988), Collar et al. (1994), and BirdLife International (2000, 2004c), plus from reviews of two species (Houbara bustard [Chlamydotis undulata] and Saker falcon [Falco cherrug]) whose categories were revised for the 2004 IUCN Red List too late to be included in BirdLife International (2004c). Information was also taken from partial assessments submitted by BirdLife to the 2002 and 2003 IUCN Red Lists (IUCN 2002, 2003). The 1988 assessment predated quantitative Red List criteria (IUCN 1994), and only the qualitative categories ‘threatened' and Near Threatened were used. Therefore, for species categorised as threatened in 1988, the category assigned in 1994 was applied to the 1988 assessment, with an appropriate category assigned for species that underwent genuine status changes during the period (see below).

#### Identifying genuine status changes between Red List assessments

Published lists of numbers of species in different Red List categories cannot simply be used to calculate the index, for several different reasons. For example, changing knowledge and taxonomy result in many category changes, but such revisions are not indicative of changes in the conservation status of species' populations. Hence, to identify those species changing categories between assessments for relevant reasons, a ‘reason for change' code was assigned for each recategorisation. These mutually exclusive codes were (1) recent genuine status change, (2) genuine status change since first assessment, (3) knowledge, (4) criteria revision, and (5) taxonomy. These last three codes were used for changes not relevant for calculating the indices.

‘Recent genuine status change' was applied to species that had undergone a genuine improvement or deterioration in status in the period since the previous assessment. This included species qualifying because of population declines (under IUCN Red List criterion A), particularly those with long generation times, for which more than half of the period during which the change occurred was subsequent to the previous assessment. This code was also applied in a few cases where species entered the Red List as a result of their elevation from subspecies to species level, but for which there had been a genuine status change when the taxon was compared to the equivalent population of the original species. For species categorised as threatened in 1988 and Critically Endangered, Endangered, or Vulnerable in 1994, genuine changes in status between these two assessments were identified by searching the account in Collar et al. (1994) and associated unpublished information for evidence of genuine status changes that had occurred in the previous 6 y.

‘Genuine status change since first assessment' was applied to species that had undergone a genuine improvement or deterioration in status in the period since the first complete assessment, but prior to the last assessment. This code denoted genuine changes in status that were not detected at the time they occurred. For example, Syrian serin (Serinus syriacus) was uplisted from Near Threatened to Vulnerable in 2004 because of the discovery that populations had declined during a drought in 1998–1999. This information was unavailable during the 2000 assessment, so the species was recategorised in 2004 and given this ‘reason for change' code (BirdLife International 2004c). For cases such as these, the Red List category for earlier assessments was back-cast using the improved understanding of earlier population sizes, trends, and ranges. This also applied to (a) extinctions that occurred after 1988; (b) species where no (or an incorrect) status change was recorded, but subsequent knowledge indicated that a genuine status change had occurred; (c) species for which an improvement in status did not immediately lead to a category revision because of the application of the ‘5-y rule' (whereby under the IUCN Red List guidelines downlisting to a lower category of threat should not occur until all of the criteria of the higher category have ceased to apply for 5 y or more; IUCN 2001; Red List Standards and Petitions Subcommittee 2003).

‘Knowledge' was applied to species recategorised owing to new information on, for example, population and range size, declines, ecological attributes, threats, or conservation efforts. This included information published or known before the last assessment, but only made available to, or discovered by, assessors since the last assessment. For example, Madagascar plover (Charadrius thoracicus) was uplisted from Near Threatened to Vulnerable in 2004 because its population was estimated to number as few as 750 individuals. However, the evidence suggests that the population may have been this small since before 1988 (BirdLife International 2004c). This code was also applied in cases where a species changed category owing to improved consistency of interpretation of information against the Red List criteria. ‘Criteria revision' was applied in cases when species changed category owing to revisions to the definitions of the IUCN Red List criteria (IUCN 2001). ‘Taxonomy' was applied in cases when species changed category owing to taxonomic ‘lumping' or ‘splitting' or for newly described species.

#### Calculating index values

The number of species in each Red List category for each complete assessment and the number of species that changed categories as a result of genuine status changes were used to determine the index value in the following way: (1) For species assessed in two consecutive assessments (i.e., excluding any listed as Not Recognised, Not Evaluated, or Data Deficient in either or both assessments), the total numbers of species in each category in the earlier assessment (excluding Extinct and Possibly Extinct, but including those species retrospectively reassigned categories owing to genuine status changes that were identified subsequently; see above) were multiplied by a weight, and these were summed to give a total score, *T,* for each assessment. The weights were set as Near Threatened = 1, Vulnerable = 2, Endangered = 3, Critically Endangered = 4, Extinct in the Wild = 5 (see below). (2) Over each period between complete assessments (1988–1994, 1994–2000, and 2000–2004) the net number of genuine changes to the total in each category was calculated, multiplied by the weights above (with Possibly Extinct and Extinct = 5), and summed to calculate the proportional change in the total score, *P.* (3) The value of the index *(I)* was set to 100 in 1988. For subsequent assessments *I* was calculated by multiplying −*P* for the previous period by the previous index value (see Table S1 for values for *T, P,* and *I* for each index).

Mathematically, the method can be described as follows, where *T* is total score; *N_c_ (t_i_)* is the number of species in category *c* at time *t_i_,* where *t_i_* is the year of the *i*th assessment (assessments are not necessarily made every year); *W_c_* is the weight for category *c; P* is proportional genuine change; *I_ti_* is the value of the index at time *t_i_; Cat(t_i_, s)* is the category of species *s* at time *t_i_; W_c_* is the weight for category *c; G_s_* = 1 if change (from *t*_(*i*−1)_ to *t_i_*) in category of species *s* is genuine (otherwise *G_s_* = 0).



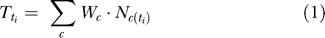





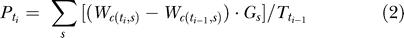





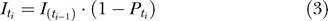



where *I_ti−1_* = 100 for the first year of assessment.

The Red List categories are ordinal ranks, whereby each step from Least Concern to Extinct indicates that at least one measure of extinction risk has become worse. The ‘equal-steps' weights listed above reflect the ordinal ranks of the categories. However, the steps between lower categories (e.g., Near Threatened to Vulnerable) translate to smaller increases in extinction risk than steps between higher categories (e.g., Endangered to Critically Endangered). Therefore we also calculated the aggregated RLI using weights based on the *relative* extinction risk associated with each category. Several of the quantitative thresholds in the Red List criteria can be used to obtain approximate values for the relative risk of extinction (for species at the lower boundary of that category). The most obvious is criterion E, which is based on quantitative analysis of extinction probability. The quantitative thresholds in criterion E change for both extinction probability and time frame for the three categories, and depend on generation length (e.g., the threshold for Endangered is a probability of extinction in the wild greater than 20% within 20 y or 5 generations). Taking a 3-generation time frame, a generation length set arbitrarily at 5 y, and assuming a constant annual risk of extinction, the 3-generation probabilities are approximately 0.5, 0.13, and 0.016 for Critically Endangered, Endangered, and Vulnerable, respectively (H. R. Akçakaya, unpublished data). However, most extinctions do not occur as a result of random catastrophes, as implied by the assumption of the constant annual risk of extinction. Many are preceded by declines, resulting in sigmoid extinction risk curves (with probability of extinction as a function of time). For such cases, the 3-generation probabilities are approximately 0.5, 0.1, and 0.025 for Critically Endangered, Endangered, and Vulnerable, respectively (see Table 3). Comparable extinction risks can also be calculated based on other Red List criteria (except A, for which there is no obvious method). Assuming that the number of mature individuals (in criteria C1 and D), range or extent of occurrence (criterion B1), and area of occupancy (criterion B2) are inversely related to risk of extinction, and fixing the risk of extinction for Critically Endangered at 0.5, it is possible to calculate the probabilities for categories Endangered and Vulnerable (see Table 3). Based on the geometric average of these estimates, the weights for Critically Endangered, Endangered, and Vulnerable are determined as 0.5, 0.05, and 0.005 (see Table 3). The weight for Extinct (and hence Extinct in the Wild and Possibly Extinct) is by definition 1.0. The weight for Near Threatened is set at 0.0005, keeping the same proportion as among the weights for the three threatened categories.

#### Calculating error bars

We calculated, using the following method, the possible range of error associated with the latest (2004) RLI value owing to time lags before genuine status changes are detected (see Discussion). We estimated how many such undetected category changes there may be for 2000–2004 using the 1994–2000 data (information gathering has improved considerably in recent years, so comparisons with time lags for the 1988–1994 period are not meaningful). In total, 128 genuine changes for 1994–2000 were identified in 2000, and an additional 17 (13.3%) were identified in the subsequent 4 y. This suggests that an additional six category changes (13.3% of 45 genuine status changes identified in 2004) may be belatedly detected for 2000–2004. We randomly selected six species from the 9,453 species that did not undergo category changes from 2000 to 2004, with a maximum of two species per category. We ran 10,000 simulations of six species moving to categories of higher extinction risk, with probabilities for each number of category steps set by the distribution of category changes for 35 ‘uplisted' species in 2000–2004. The maximum value for *P* (proportional genuine change) from these simulations gave the lower error bar for the 2004 RLI value. Similarly, we ran 10,000 simulations of six species moving to categories of lower extinction risk (with probabilities for each number of category steps set by the distribution of category changes for ten ‘downlisted' species in 2000–2004), and took the minimum value for *P* to give the upper error bar. These are very similar to the minimum and maximum values for *P* derived by simulating an additional six species moving in a direction (and by a number of categories) in proportion to the distribution of these values for all 45 species that underwent genuine status changes in 2000–2004 (see Table S2).

#### Disaggregating indices

One of the purposes of the RLIs is to illustrate trends over time in the threat status of species in different biogeographic realms, ecosystems and families or species groups. Species were assigned (based on native distributions, excluding cases of vagrancy) to one or more biogeographic realms (Palearctic, Afrotropical, Indo-Malayan, Nearctic, Neotropical, and Australasian/Oceanic) following the boundaries mapped by Newton (2003) except that Australasian was pooled with Oceanic, and Antarctic was excluded. Where a species was assigned to more than one realm, it was included in calculating the score *(T)* for each of those realms. This is because a species could potentially undergo genuine changes in status in any or all realms in which it occurs. However, so that trends in indices for particular realms reflect changes in the threatening processes operating within each particular realm (rather than threats operating elsewhere in the range of the species), species were only included in the calculation of *P* for a particular realm if the genuine status change had been driven by factors operating in that realm. For example, Saker falcon *(F. cherrug)* occurs in the Palearctic, Indo-Malayan, and Afrotropical realms and was included in the score calculations for each of these. However, recent declines have been driven by factors operating on the breeding grounds in Central Asia (Environmental Research and Wildlife Development Agency, unpublished data; BirdLife International 2004c), so the genuine change was only calculated for the Palearctic realm. By contrast, the black-browed albatross *(Thalassarche melanophrys)* has declined as a result of incidental capture in commercial longline fisheries in oceans in the Afrotropical, Neotropical, and Australasian/Oceanic realms (Robertson and Gales 1998; BirdLife International 2004c), and so this genuine change was incorporated into the calculation of *P* for all three realms.

The index was disaggregated for ecosystem (terrestrial, marine, and freshwater) and for two terrestrial habitat types (forest and shrubland/grassland; see below) in a similar way. Species were included in the calculation of *T* for all ecosystems and habitats for which they were scored, but only included in the calculation of *P* for a particular ecosystem or habitat if the genuine status change had been driven by threatening processes operating in that ecosystem or habitat. Species were only assigned to a habitat type if this was of critical or major importance (i.e., the species typically occurs in no other habitat, or just one other habitat at some point in its life cycle).

To exemplify how the approach can be used for taxonomic subsets of species, RLIs were also calculated for several high-profile species groups with specific conservation interest groups: raptors (Falconiformes), game birds (Galliformes), and parrots (Psittaciformes); and for species groups relevant to particular international conservation treaties: waterbirds (as listed in Wetlands International 2003) covered by the Ramsar convention, migrant species covered by the CMS, and albatrosses (Diomedeidae) and large petrels (*Macronectes* spp. and *Procellaria* spp.) covered by the ACAP under the CMS.

Sample sizes in the figure legends give, for the subset of species plotted, the total number of category changes owing to genuine status changes during 1988–2004 (but note that a small number of species underwent genuine status changes in more than one period between assessments) and the total number of species in categories Extinct in the Wild, Critically Endangered, Endangered, Vulnerable, and Near Threatened in at least one assessment during the period (and that are taxonomically recognised at present).

## Supporting Information

Table S1 Index Values: Values for *T, P,* and *I* for Each Period and for Each Index (66 KB DOC).

Table S2 Calculating Error Bars: Simulated *P-*Values (Proportional Change in the Index Score *T*) Used to Determine Error Bars for 2004 RLI Value Lists the simulated *p-*values (proportional change in the index score *T*) based on the assumption that an additional six genuine changes occurred from 2000 to 2004 but have not yet been identified owing to time lags in knowledge (see Materials and Methods). Six species were randomly selected from those that did not change category from 2000 to 2004 (*n* = 9,453 species), with a maximum of two species from each category. For each species, the number and direction of category changes were randomly assigned with probabilities (1) based on the change in categories for the ten species that underwent genuine status changes and were downlisted to a lower category of threat during 2000–2004 (‘only down'); (2) based only on the 35 species that were uplisted to a higher category of threat (‘only up'); (3) based on all 45 species (‘both up and down'); and (4) based on all 45 species with probabilities that were set individually for each threat category (‘category dependent,' so that, e.g., the probability of an Least Concern species being downlisted to a lower category of threat was zero). In each case, the procedure was repeated 10,000 times to calculate the minimum, maximum, mean, and standard deviation of the simulated *p-*value.
The upper error bars for the RLI were determined by the minimum simulated *p-*value for cases when all six species were downlisted to lower categories of threat (shown in red in the table). The lower error bars were determined by the maximum simulated *p-*value for cases when all six species were uplisted to higher categories of threat (shown in blue in the table). In both cases the values are close to those calculated if the six species changed category, with probabilities based on the direction and number of category changes for all 45 species, and encompass those derived using the method based on category-dependent probabilities.
(35 KB DOC).

## Abbreviations

ACAP: Agreement on the Conservation of Albatrosses and Petrels
CBD: Convention on Biological Diversity
CMS: Convention on Migratory Species
IUCN: World Conservation Union
RLI: Red List Index
