# Supplementary material for: Measuring Global Trends in the Status of Biodiversity: Red List Indices for Birds
Source: PLoS Biol. 2004 Oct 26;2(12):e383. doi: 10.1371/journal.pbio.0020383 (PMC524254; doi:10.1371/journal.pbio.0020383)
Supplement: Table S1 — (66 KB DOC). [file pbio.0020383.st001.doc]

**Table S1**. Index Values: Values for *T,* *P,* and *I* for Each Period and for Each Index

| Index | 1988-1994 | | | 1994-2000 | | | 2000-2004 | | |
| --- | --- | --- | --- | --- | --- | --- | --- | --- | --- |
|  | *T* | *P* | *I* | *T* | *P* | *I* | *T* | *P* | *I* |
| All species | 2903 | 0.0214 | 97.864 | 3606 | 0.0383 | 94.119 | 3772 | 0.0093 | 93.246 |
| All species (weighted by extinction risk) | 87.98 | 0.0784 | 92.161 | 99.38 | 0.0688 | 85.816 | 109.97 | 0.0088 | 85.065 |
| Afrotropical realm | 540 | 0.0259 | 97.407 | 624 | 0.0208 | 95.378 | 659 | 0.0106 | 94.365 |
| Indomalay realm | 583 | 0.0223 | 97.770 | 770 | 0.1156 | 86.469 | 758 | -0.0013 | 86.584 |
| Nearctic realm | 94 | 0.0426 | 95.745 | 115 | 0.0435 | 91.582 | 144 | 0.0139 | 90.310 |
| Neotropical realm | 1046 | 0.0143 | 98.566 | 1238 | 0.0113 | 97.451 | 1357 | 0.0140 | 96.087 |
| Australasian/Oceanic realm | 605 | 0.0413 | 95.868 | 842 | 0.0190 | 94.046 | 946 | 0.0201 | 92.157 |
| Palearctic realm | 291 | 0.0275 | 97.251 | 358 | 0.0363 | 93.719 | 347 | 0.0288 | 91.019 |
| Terrestrial ecosystem | 2691 | 0.0230 | 97.696 | 3364 | 0.0375 | 94.037 | 3525 | 0.0060 | 93.477 |
| Freshwater ecosystem | 307 | 0.0521 | 94.788 | 362 | 0.0166 | 93.217 | 373 | 0.0134 | 91.968 |
| Marine ecosystem | 126 | 0.0159 | 98.413 | 187 | 0.0428 | 94.203 | 264 | 0.0341 | 90.991 |
| Forest | 1948 | 0.0185 | 98.152 | 2420 | 0.0479 | 93.447 | 2683 | 0.0056 | 92.925 |
| Shrubland/grassland | 596 | 0.0235 | 97.651 | 742 | 0.0202 | 95.677 | 806 | 0.0050 | 95.202 |
| Raptors | 107 | 0.0000 | 100.00 | 123 | 0.0488 | 95.122 | 128 | 0.0156 | 93.636 |
| Gamebirds | 186 | 0.0161 | 98.387 | 210 | 0.0286 | 95.576 | 213 | 0.0094 | 94.679 |
| Parrots | 200 | 0.0600 | 94.000 | 270 | 0.0111 | 92.956 | 282 | 0.0000 | 92.956 |
| Migrants (CMS) | 356 | 0.0478 | 95.225 | 447 | 0.0336 | 92.029 | 498 | 0.0321 | 89.072 |
| Waterbirds (Ramsar) | 336 | 0.0506 | 94.940 | 403 | 0.0124 | 93.763 | 409 | 0.0147 | 92.387 |
| Albatrosses and petrels (ACAP) | 102 | 0.0490 | 95.098 | 152 | 0.0724 | 88.216 | 239 | 0.0377 | 84.894 |

In Table 2, for each period the *T* value for the start year, *P* value (proportional change in *T*) over the period, and *I* (RLI value) for the end year are given for each index. Note that *I* is set to 100 in 1988 for all indices.
