# Supplementary material for: Measuring Global Trends in the Status of Biodiversity: Red List Indices for Birds
Source: PLoS Biol. 2004 Oct 26;2(12):e383. doi: 10.1371/journal.pbio.0020383 (PMC524254; doi:10.1371/journal.pbio.0020383)
Supplement: Table S2 — Lists the simulated p-values (proportional change in the index score T) based on the assumption that an additional six genuine changes occurred from 2000 to 2004 but have not yet been identified owing to time lags in knowledge (see Materials and Methods). Six species were randomly selected from those that did not change category from 2000 to 2004 (n = 9,453 species), with a maximum of two species from each category. For each species, the number and direction of category changes were randomly assigned with probabilities (1) based on the change in categories for the ten species that underwent genuine status changes and were downlisted to a lower category of threat during 2000–2004 (‘only down'); (2) based only on the 35 species that were uplisted to a higher category of threat (‘only up'); (3) based on all 45 species (‘both up and down'); and (4) based on all 45 species with probabilities that were set individually for each threat category (‘category dependent,' so that, e.g., the probability of an Least Concern species being downlisted to a lower category of threat was zero). In each case, the procedure was repeated 10,000 times to calculate the minimum, maximum, mean, and standard deviation of the simulated p-value. The upper error bars for the RLI were determined by the minimum simulated p-value for cases when all six species were downlisted to lower categories of threat (shown in red in the table). The lower error bars were determined by the maximum simulated p-value for cases when all six species were uplisted to higher categories of threat (shown in blue in the table). In both cases the values are close to those calculated if the six species changed category, with probabilities based on the direction and number of category changes for all 45 species, and encompass those derived using the method based on category-dependent probabilities. (35 KB DOC). [file pbio.0020383.st002.doc]

***Table S2****. Calculating Error Bars: Simulated P Values (Proportional Change in the Index Score T) Used to Determine Error Bars for 2004 RLI Value*

| Weighting | Direction of simulated changes | Current *P* (2000-2004) | Simulated *P* (2000-2004) | | | |
| --- | --- | --- | --- | --- | --- | --- |
|  |  |  | Max. | Min. | Mean | St.Dev. |
| Equal Step |  | 0.009279 |  |  |  |  |
|  | Only up |  | 0.0130 | 0.0108 | 0.0114 | 0.0004 |
|  | Only down |  | 0.0082 | 0.0071 | 0.0080 | 0.0002 |
|  | Both up and down |  | 0.0132 | 0.0079 | 0.0106 | 0.0007 |
|  | Category dependent |  | 0.0130 | 0.0085 | 0.0110 | 0.0006 |
| Extinction Risk |  | 0.008753 |  |  |  |  |
|  | Only up |  | 0.0396 | 0.0097 | 0.0179 | 0.0049 |
|  | Only down |  | 0.0087 | -0.0011 | 0.0068 | 0.0023 |
|  | Both up and down |  | 0.0395 | 0.0002 | 0.0154 | 0.0052 |
|  | Category dependent |  | 0.0272 | -0.0002 | 0.0117 | 0.0042 |
